# 疏肝肺积方结合心理干预对原发性肺癌患者抑郁、焦虑的影响

**DOI:** 10.3779/j.issn.1009-3419.2012.04.04

**Published:** 2012-04-20

**Authors:** 逸临 姚, 和根 李, 苓霜 刘, 丽红 赵, 玲 许, 建立 孙

**Affiliations:** 200032 上海，上海中医药大学附属龙华医院肿瘤科 Department of Medical Oncology, Longhua Hospital Affiliated to Shanghai University of Traditional Chinese Medicine, Shanghai 200032, China

**Keywords:** 肺肿瘤, 疏肝肺积方, 心理干预, 抑郁, 焦虑, Primary lung cancer, Feiji Decoction for soothing the liver, Psychotherapy, Depression, Anxiety

## Abstract

**背景与目的:**

肺癌是我国最常见的恶性肿瘤之一，在治疗中给患者带来了巨大的躯体病痛和情绪障碍，本实验旨在研究疏肝肺积方结合心理干预对原发性肺癌患者抑郁情绪和焦虑情绪的影响，并进一步探讨患者抑郁和焦虑之间的相互关系。

**方法:**

将118例原发性非小细胞肺癌患者随机分为两组：疏肝肺积方结合心理干预加化疗组（联合治疗组57例）和单纯化疗组（61例）。联合治疗组采用疏肝肺积方和心理干预与化疗的联合应用；单纯化疗组仅接受化疗，共完成两次化疗，为一个总疗程。采用ZUNG抑郁自评量表和ZUNG焦虑自评量表测定心理状况。

**结果:**

联合治疗组的抑郁、焦虑评分治疗后较治疗前下降（*P* < 0.01）；单纯化疗组的抑郁、焦虑评分治疗后较治疗前上升（*P* < 0.01）；联合治疗组的抑郁、焦虑评分比单纯化疗组患者明显下降（*P* < 0.01）；患者抑郁与焦虑指数呈正相关（*P* < 0.01）。

**结论:**

疏肝肺积方结合心理干预能缓解并改善肺癌患者的抑郁情绪和焦虑情绪，具有较好的临床疗效。

## Abstract (en)

**Background and objective:**

Lung cancer is the most common type among all malignant tumors, bringing huge pain and mood disorders to patients suffering from it. This study aims to evaluate the effect of the Feiji Decoction for soothing the liver combined with psychotherapy for depression and anxiety on patients with primary lung cancer. It also aims to further explore the relationship between depression and anxiety.

**Methods:**

A total of 118 patients with primary non-small cell lung cancer were randomly divided into two groups. The combined therapy group consisted of 57 patients who were treated with Feiji Decoction for soothing the liver and psychotherapy combined with chemotherapy. The control group consisted of 61 patients who were treated with chemotherapy only. Both groups were observed for two treatment courses. The Zung Self-reported Depression Scales (SDS) and Zung Self-reported Anxiety Scales (SAS) were used to assess the patients' psychology status in both groups before and after treatment scales.

**Results:**

After treatment, the depression and anxiety scores of the therapy group remarkably decreased compared with scores before treatment, indicating a statistical significance (*P* < 0.01). In comparison, after treatment, the depression and anxiety scores of the control group significanfly increased compared with scores before treatment, indicating a statistical significance (*P* < 0.01) as well. The depression and anxiety scores of the therapy group significanfly decreased compared with scores of the control group, showing statistical significance (*P* < 0.01). Thus, depression and anxiety score has a close co-relationship, showing significant positive correlation (*P* < 0.01).

**Conclusion:**

Feiji Decoction for soothing the liver combined with psychotherapy can alleviate depression and anxiety in patients with primary lung cancer. This therapy has good clinical therapeutic effect.

肺癌是我国最常见的恶性肿瘤之一，2/3的患者在确诊时已属中晚期肺癌，在治疗中给患者带来了巨大的躯体病痛和情绪障碍，诸如焦虑和抑郁等，这不仅会引起患者生存质量的下降，而且足以导致其生物学改变。抑郁是肿瘤患者的常见症状，是一种情绪和生命力都低至绝望的心理紊乱状态。抑郁者常常性格孤僻，对生活失去兴趣，社会活动减少，躲避他人，还可能有疼痛不适、心悸、视力模糊、口干、头痛和恶心等不适主诉，严重者有自杀倾向^[1]^。焦虑是指以广泛和持续性焦虑或反复发作的惊恐不安为主要特征的神经症性障碍，患者的焦虑与惊恐并非由实际威胁或危险所引起，或其紧张不安与惊恐程度与现实处境不相称，经检查证实并没有相应的器质性基础。ZUNG抑郁自评量表（Self-reported Depression Scales, SDS）和ZUNG焦虑自评量表（Self-reported Anxiety Scales, SAS）为短程自评量表，具有很高的内在一致性，简便且易于操作，可用于肿瘤患者抑郁障碍和焦虑障碍的评估和随访^[2]^。本研究采用SDS和SAS作为观察指标，对疏肝肺积方结合心理干预治疗原发性支气管肺癌患者的情绪障碍进行观察，为研究心理社会因素在肿瘤防治过程中的作用提供思路和方法，对综合有效的治疗癌症、提高患者生存质量、延长生存期提供重要的临床资料。

## 1 资料与方法

### 1.1 病例选择标准

#### 1.1.1 纳入标准

① 符合原发性支气管肺癌诊断标准^[3]^和原发性支气管肺癌分期标准^[4]^；②经临床、影像学、细胞学和病理学检查确诊的肺癌患者；③年龄在18岁-75岁之间；④准备进行化疗的肺癌患者；⑤预计生存期≥6个月；⑥KPS≥60分；⑦无严重肝肾功能损害、无智力障碍；⑧小学及小学以上文化程度；⑨愿意加入研究者。

#### 1.1.2 排除标准

① 既往和目前有精神疾病和精神疾病家族史；②有智力或认知功能障碍患者；③有精神疾病药物或酒精依赖史；④伴有严重的并发症；⑤伴有其它系统严重疾病需要专科治疗者；⑥依从性差。

#### 1.1.3 剔除标准

① 治疗中出现严重并发症或病情急剧恶化需采用紧急处理措施；②由于病情需要暂停化疗、连续化疗未完成两周期；③由于各种原因未能完成疏肝肺积方结合心理干预及资料填写不全。

### 1.2 一般资料

本研究共入组140例原发性非小细胞肺癌患者，全部病例来源于上海中医药大学附属龙华医院及上海交通大学附属胸科医院。依据随机数字表将患者随机分为疏肝肺积方结合心理干预加化疗组和单纯化疗组。最终完成两次调查的病例为118例，包括联合治疗组57例和单纯化疗组61例，22例由于各种原因脱落，完成率84.3%。对两组患者治疗前的一般情况包括性别、年龄、婚姻状况、文化程度、职业、治疗费用、临床分期、病理类型进行检验，两组差异无统计学意义（*P* > 0.05）。

### 1.3 治疗方法

联合治疗组患者同时接受疏肝肺积方结合心理干预与化疗，单纯化疗组患者仅接受化疗，两组患者都给予必要的对症治疗，疼痛者按照WHO三阶梯原则给药；骨髓抑制达Ⅱ度及以上者，予重组人粒集落细胞刺激因子（granulocyte colony-stimulating factor, G-CSF）至恢复正常，化疗前予以必要的止吐药物。

#### 1.3.1 疏肝肺积方结合心理干预治疗

自患者入组化疗前3天，开始服用中药并进行心理干预，直至第二次化疗的第28天，为一个总疗程。

##### 1.3.1.1 中药

以疏肝肺积方为基本方（生黄芪30 g，生白术15 g，北沙参15 g，石上柏30 g，七叶一枝花24 g，冰球子30 g，山萸肉12 g，仙灵脾15 g，玫瑰花9 g，八月札15 g，绿萼梅9 g），可酌情加减。

##### 1.3.1.2 心理干预

① 心理诱导教育：肿瘤相关知识，常见治疗（手术、放疗、化疗）的不良反应，有效病例的介绍；②行为训练：每日练习太极拳一次，约15 min；或进行放松训练、引导性想象一次，约15 min；③个体化心理治疗：因人而异施治，针对患者出现的不良心理表现如焦虑、抑郁等进行个别交谈，帮助患者认识问题，改善焦虑或抑郁情绪；④家庭成员配合治疗：向联合治疗组家属说明综合心理干预措施的意义，组织患者与家属共同面对疾病，分担不良情绪，并改善患者及家属的心理状态。

#### 1.3.2 化疗

采用NP方案：长春瑞宾25 mg/m^2^ d1, d8；顺铂75 mg/m^2^；或者GP方案：吉西他滨1, 000 mg/m^2^ d1, d8, d15；顺铂75 mg/m^2^；每28天为1个周期，两个化疗周期为1个总疗程。

### 1.4 观察指标及疗效评定

#### 1.4.1 SDS

入组化疗前3天测定一次，第二次化疗的第28天测定一次。由患者进行填写。每个条目均按4个等级，评分为1分-4分。20个条目中有10项是用负性词陈述的，按上述1-4顺序评分；其余10项是用正性词陈述的，按4-1顺序反向评分。将20个条目的各个得分相加，即得到粗分，用粗分乘以1.25以后取其整数部分，就得到标准分。分数越高，抑郁症状越重。治疗后较治疗前评分下降为抑郁改善，治疗后较治疗前评分上升为抑郁加重。

抑郁严重度指数=粗分/80。指数 < 0.5者为无抑郁；0.50-0.59者为轻度抑郁；0.60-0.69者为中度抑郁； > 0.7者为重度抑郁。

#### 1.4.2 SAS

入组化疗前3天测定一次，第二次化疗的第28天测定一次。由患者进行填写。每个条目均按4个等级，评分为1分-4分。20个条目中有10项是用负性词陈述的，按上述1-4顺序评分；其余5项是用正性词陈述的，按4-1顺序反向评分。将20个条目的各个得分相加，即得到粗分，用粗分乘以1.25以后取其整数部分，就得到标准分。分数越高，焦虑症状越重。治疗后较治疗前评分下降为焦虑改善，治疗后较治疗前评分上升为焦虑加重。

焦虑严重度指数=粗分/80。指数在 < 0.5者为无焦虑；0.50-0.59者为轻度焦虑；0.60-0.69者为中度焦虑； > 0.7者为重度焦虑。

### 1.5 统计学方法

采用SPSS 13.0统计软件包建立数据库，并进行统计学处理。计量资料经正态性检验及方差齐性检验后，如果服从正态分布及方差齐，采用*t*检验或*F*检验；如果不服从正态分布或方差不齐，则采用秩合检验。计数资料的比较采用卡方检验或*Fisher's*精确概率法，*P* < 0.05为差异有统计学意义。

## 2 结果

### 2.1 SDS

#### 2.1.1 联合治疗组和单纯化疗组患者治疗前后抑郁病例分布情况

由表 1可见：联合治疗组患者治疗后较治疗前抑郁程度呈下降趋势；单纯化疗组患者治疗后较治疗前抑郁程度呈上升趋势。

**1:** 联合治疗组和单纯化疗组患者治疗前后抑郁病例分布情况 The distribution of patients with depression before and after-treatment of combination-therapy group and chemotherapy group

Grade	Combination-therapy group			Chemotherapy group	
	Before-treatment	After-treatment		Before-treatment	After-treatment
Normal	19	44		26	17
Mild	21	10		21	17
Moderate	17	2		14	12
Severe	0	1		0	15
Total	57	57		61	61

#### 2.1.2 联合治疗组和单纯化疗组患者治疗前后抑郁标准分及自身对照

联合治疗组患者治疗后的抑郁评分与治疗前相比明显下降（42.85±9.58 *vs* 55.00±12.31, *t*=7.563, *P* < 0.01），单纯化疗组患者治疗后的抑郁评分与治疗前相比明显上升（59.88±13.57 *vs* 50.94±11.37, *t*=6.767, *P* < 0.01），表明联合治疗组患者的抑郁情绪在治疗后明显改善，单纯化疗组患者的抑郁情绪在治疗后较治疗前明显加重。

#### 2.1.3 联合治疗组和单纯化疗组患者治疗前后抑郁标准分的组间比较

联合治疗组和单纯化疗组患者治疗前的抑郁评分无统计学差异（55.00±12.31 *vs* 50.94±11.37, *t*=1.861, *P* > 0.05）；联合治疗组患者治疗后的抑郁评分比单纯化疗组明显下降（42.85±9.58 *vs* 59.88±13.57, *t*=7.914, *P* < 0.01），与单纯化疗组相比，联合治疗组患者治疗后的抑郁情绪明显改善。

### 2.2 SAS

#### 2.2.1 联合治疗组和单纯化疗组患者治疗前后焦虑病例分布情况

由表 2可见：联合治疗组患者治疗后较治疗前焦虑程度呈下降趋势；单纯化疗组治疗后较治疗前焦虑程度呈上升趋势。

**2:** 联合治疗组和单纯化疗组患者治疗前后焦虑病例分布情况 The distribution of patients with anxiety before and after-treatment of combination-therapy group and chemotherapy group

Grade	Combination-therapy group			Chemotherapy group	
	Before-treatment	After-treatment		Before-treatment	After-treatment
Normal	33	49		43	31
Mild	16	6		12	10
Moderate	7	2		5	13
Severe	1	0		1	7
Total	57	57		61	61

#### 2.2.2 联合治疗组和单纯化疗组患者治疗前后焦虑标准分及自身对照

联合治疗组患者治疗后的焦虑评分与治疗前相比明显下降（37.98±8.39 *vs* 47.63±10.00, *t*=7.545, *P* < 0.01），单纯化疗组患者治疗后的焦虑评分与治疗前相比明显上升（51.95±12.56 *vs* 44.51±10.22, *t*=7.006, *P* < 0.01），表明联合治疗组患者的焦虑情绪治疗后较治疗前明显改善，单纯化疗组患者的焦虑情绪治疗后较治疗前明显加重。

#### 2.2.3 联合治疗组和单纯化疗组患者治疗前后焦虑标准分的组间比较

联合治疗组和单纯化疗组患者治疗前的焦虑评分无统计学差异（47.63±10.00 *vs* 44.51±10.22, *t*=1.676, *P* > 0.05）；联合治疗组患者治疗后的焦虑评分比单纯化疗组明显下降（37.98±8.39 *vs* 51.95±12.56, *t*=6.194, *P* < 0.01），与单纯化疗组相比，联合治疗组患者治疗后的焦虑情绪明显改善。

### 2.3 患者抑郁指数与焦虑指数的相关性分析

呈正相关（*r*=0.582, *P* < 0.01）。

## 3 讨论

适当的心理社会干预对患者的恢复是有益的，它可以改善个体的应付能力，减少情绪上的烦恼和情感上的孤独，从而改善患者的心理功能，以及在一定程度上缓解症状，最终提高其生存质量，甚至延长个体的存活时间。心理社会因素在肿瘤的发生、发展和转归中起着重要的作用，因而及时发现并重视肿瘤患者的异常心理状况并对他们实施心理干预治疗应成为肿瘤综合治疗的一部分。

中医把人的心理思维活动的产生归属特定的脏腑，《素问·阴阳应象大论》曰：“人有五脏化五气，以生喜怒悲忧恐”。因而，中医认为人的情志思维活动会影响脏腑气血功能的活动。情志致病学说是中医病因学理论体系“三因学说”的重要组成部分，情志致病是诸多心身疾病和隐性病理状态形成和维持的重要因素，这在肿瘤的病因病机中体现尤为明显。王肯堂在《医学津梁》中也曰：“忧郁不解，思虑太过，忿怒不伸，惊恐变故，以致内气并结与上焦而噎嗝之疾成矣”。《妇人良方》中认为乳岩（指现代医学的乳腺癌）的发生“此属肝脾郁怒，气血亏损”。《外科正宗》中认为：“忧郁伤肝，思虑伤脾，积想有心，所愿不得志者，致经络疲惫，聚结成核”。由此可见，情志表现过度、七情内伤必然会导致脏腑功能失调，气机升降不畅，久则脏腑亏虚，气滞血瘀，痰凝毒结，癌瘤形成。

古人所描述的情志与肿瘤致病的关系与现代肿瘤病因学中的心理应激和情绪障碍是一致的，心理神经内分泌学重视心理因素在疾病发生中的作用，认为应激能够增加病毒感染，抑制免疫，导致癌症。恶性肿瘤患者躯体上承受着疾病、手术和放、化疗所带来的病痛，心理上更要承受对疾病的困惑、对死亡的恐惧以及对家庭其它成员的愧疚和对经济开销的估算等许多思想负担。身患肿瘤对他们而言是躯体和心理上的双重应激，这些应激都可理解为危险信号，如果此时患者不能采取积极的应对风格，过度夸大危险性而低估自身的应对能力就容易产生抑郁、焦虑、述情障碍等表现，从而影响到免疫、内分泌、中枢神经系统等正常运作，直接干扰肿瘤预后及患者的躯体症状，生存质量明显下降。Savard^[5]^发现，抑郁与T细胞CD8^+^明显相关。Augustin^[6]^发现，焦虑与CD56^+^变化明显相关，在女性患者中更明显，但与年龄无关。李建中等^[7]^研究结果显示肿瘤患者CD3^+^水平除与社会支持总分呈正相关外，与性别、年龄、艾森克量表E分、N分、HAMD总分及不成熟防御机制因子分均呈负相关。尤以女性、高龄、脾气躁、易冲动、心境变换剧烈及情绪抑郁者体内CD3^+^水平低下，其中情绪抑郁对CD3^+^影响最大。王卉等^[8]^发现肺癌患者的SDS、SAS分数越高其CD4/CD8就越低，也就是说患者的免疫功能与抑郁焦虑之间存在负相关。魏永长等^[9]^研究表明积极应对和CD56与生活质量明显正相关；消极应对与CD56明显负相关；抑郁与淋巴细胞数明显正相关；T细胞亚群和自然杀伤细胞明显降低，表明焦虑和抑郁可降低机体免疫功能。

基于以上理论，本研究将疏肝肺积方与心理干预措施相结合，运用于原发性支气管肺癌患者，对治疗后患者的情绪障碍抑郁和焦虑评分进行疗效评价。结果表明：通过对患者实施心理干预措施并结合运用疏肝肺积方后，联合治疗组患者的抑郁发生率由66.7%下降至22.8%，焦虑发生率由42.1%下降至14%；而单纯化疗组患者在经过两次化疗后抑郁发生率由57.4%上升至72.1%，其中重度抑郁的发生率为24.6%；单纯化疗组焦虑发生率由29.5%上升至49.2%，重度焦虑的发生率为11.5%。抑郁、焦虑的加重与化疗给患者带来的诸多副反应，比如恶心呕吐、疲倦、脱发、骨髓抑制、免疫力低下等，都是密切相关的。李桢等^[10]^对61例围手术期癌症患者进行支持治疗、患者互助支持和家庭社会支持等治疗，结果表明心理干预能明显改善其抑郁、焦虑、恐惧等情绪，有助于躯体疾病的治疗及生存质量的提高。倪秉强等^[11]^对热化疗肿瘤患者进行心理行为干预，结果发现干预组的焦虑、抑郁心理状态较对照组明显改善，缓解了负性情绪，从而提高了患者的生活质量。值得注意的是，联合治疗组患者治疗后SDS分值仍稍高于正常人群，这提示通过服用疏肝肺积方和采取心理行为干预措施能在一定程度上减轻肺癌患者的抑郁情绪，但肺癌作为一个严重的不良应激事件，其所导致的生存期的缩短、疾病的复发或转移以及在治疗中造成的家庭经济困难等，诸多因素给患者所带来的负性情绪是很难完全消除的。研究中还发现，患者的焦虑情绪与对肺癌知识的缺乏、对生命的担忧以及对预后的顾虑有关。肿瘤患者除本身具有特质性焦虑以外，多数还会出现反应性焦虑。这种焦虑主要是由于治疗过程中伴随出现的各种不适症状，或是外表形象受损比如脱发、憔悴以及各种不确定因素所致。化疗是肿瘤治疗的主要手段，常导致患者焦虑不安、恐惧紧张^[12]^；治疗后，又出现近期和远期的毒性反应，这些毒副作用可引起患者直接和间接的心理应激反应。

由此可见，疏肝肺积方结合心理干预能明显缓解肺癌患者的抑郁和焦虑情绪，激发患者的生存欲望，增强患者忍受治疗痛苦的耐受力，增加患者对治疗的依从性，消除由于心理原因带来的躯体不适，并帮助患者尽快从化疗的不适感和抑郁焦虑感中恢复过来，从而明显降低患者抑郁、焦虑的发生率和程度，有利于提高患者的生存质量。因此，在今后的工作中需要加强心理干预的力度，培养患者良好的应付策略和治疗态度，以更好地维护肿瘤患者的生活质量。
